# Role of microsurgical free flap reconstruction in managing complex wound: a retrospective cross-sectional study

**DOI:** 10.11604/pamj.2022.43.211.36595

**Published:** 2022-12-30

**Authors:** Abdulfattah Altam, Ahmed Alsaaidi, Waleed Aljbri, Faisal Ahmed, Saleh Al-Wageeh, Qasem Alyhari, Burkan Nasr, Abdullah Al-Naggar, Mohamed Badheeb, Ebrahim Al-Shami

**Affiliations:** 1 Department of General Surgery, School of Medicine, 21 September University, Sana'a, Yemen,; 2 Department of General Surgery, Al-Thora Modern General Hospital, Sana´a, Yemen,; 3 Department of Urology, School of Medicine, 21 September University, Sana'a, Yemen,; 4 Department of Urology, School of Medicine, Ibb University of Medical Sciences, Ibb, Yemen,; 5 Department of General Surgery, School of Medicine, Ibb University of Medical Sciences, Ibb, Yemen,; 6 Department of Anesthesiology, Al-Thora Modern Hospital, Faculty of Medicine, Sana´a University of Medical Sciences, Sana´a, Yemen,; 7 Department of Internal Medicine, College of Medicine, Hadhramaut University, Mukalla, Yemen

**Keywords:** Microsurgery, free flaps, complex wounds, microvascular

## Abstract

**Introduction:**

while reconstruction of complex wounds with severe tissue defects has been a significant problem in plastic surgery, free flap microsurgical procedures could solve many of these problems. In Yemen, data regarding free flap microsurgery for complex wounds are scarce. This study aimed to share our microsurgery experiences in repairing complex wounds using different free flaps in a resource-limited setting.

**Methods:**

a retrospective cross-sectional study between April 2019 and June 2022 conducted at 21 University-affiliated hospitals included 30 patients with complex wound defects that were not amenable for regional, pedicle procedures, or skin grafts and underwent microsurgical reconstructions with deferent free flap tissue transfer. The primary outcome was flap survival or failure, while the secondary outcome was postoperative complications.

**Results:**

the main age was 34.76 ± 16.88 years, with 24 (80%) males and 6 (20%) females. Replacing extensive tissue loss caused by road traffic accidents was the most common indication (36.6%). The mean defects required to be reconstructed were 84.9 ± 44.70 cm^2^. The lower extremities accounted for the majority of reconstructed defects (50%), and mostly (23.3%) involved the leg. Only 10 (33.3%) flaps were performed immediately within 48 hours of trauma. The fibulae osteo-cutaneous free flap (30.0%), radial forearms free flap (23.3%), and anterolateral thigh flap (23.3%) were used most commonly. All flaps were harvested and repaired under loupe magnification or operative microscope by a single surgeon. The overall flap success rate was 83.3%. The total complication rate was 23.3%, and postoperative infection and partial flap necrosis occurred in 3 (10.0%) and 2 (6.6%) patients, respectively. A total flap loss occurred in 5 (16.7%) patients.

**Conclusion:**

reconstruction of complex wounds with microsurgical free flaps is a viable option even in a resource-limited setting. In our study, microsurgery with fibulae osteo-cutaneous free flap was the most commonly used. Despite many limitations, microsurgical free flaps were effective in treating individuals operated on in our setup with a limb salvage rate of 83.3%.

## Introduction

Traumatic soft tissue injuries are frequently associated with complex tissue loss and infection. Conventional treatment for these patients requires various operative procedures and prolonged hospital stays, resulting in poor functional recovery [1,2]. With a better understanding of wound care and improved competency in the microvascular free tissue flap transfer approach in managing damaged tissue, the management of such complicated wounds has improved significantly in recent years [3,4]. Reconstruction aims to maximize functional and esthetic outcomes, minimize donor damage, and prevent infections while having sufficient soft tissue coverage for critical organs [3,5].

To provide adequate soft tissue coverage in these cases, free flap tissue transfer is required. Many flaps, including radial forearms, latissimus dorsi, anterolateral thigh flap, fibulae osteo-cutaneous, and gracilis-free flaps, can be used to cover wounds [4,6,7]. Decision-making regarding the flap type is often influenced by institutional practices, facilities, and proficiency. Microsurgical free muscle flap transfer necessitates more skill, facilities, and intensive monitoring than fasciocutaneous flaps. However, this technique has become more common as more specialists are involved in reconstructive microsurgery [4,8]. Studies on microsurgical free flap reconstruction and its consequences are quite sparse in our region due to inadequate resources despite their importance. For instance, Altam *et al*. reported a great outcome of microsurgical reconstruction provided great outcomes of the enormous traumatic oromandibular defect by osteocutaneous fibula-free flap in a 9-year-old [9]. We believe this study will add a wider view of microsurgery experiences in a data-limited nation, where complex wound defects are highly common. This study aimed to share our microsurgery experience using different free flaps to repair difficult and complex wounds in resource-limited settings.

## Methods

**Study design:** a retrospective cross-sectional study between April 2019 and June 2022 conducted at 21 University-affiliated hospitals included 30 consecutive patients with complex and challenging wound defects that were not amenable for regional, pedicle, or skin grafts and underwent microsurgical reconstructions with the transfer of the different free flaps. All flap transfers were performed by the same surgeon (Professor A. Altam).

**Inclusion criteria:** all patients aged more than 10 years with complex and difficult wound defects that were not amenable to regional, pedicle procedures, or skin grafts underwent microsurgical reconstructions with deferent free tissue transfer in our center.

**Exclusion criteria:** patients treated in other centers, pregnant patients, and patients who did not have a complete clinical history were excluded.

**Data collection:** the demographic characteristics of the patients, such as age, gender, cause of a defect, size of the defect (cm^2^), location of the defect, flap usage, operative time, complications, follow-up time, and outcome, were gathered and analyzed.

**Preoperative assessment:** all patients underwent preoperative clinical evaluation, including; history, physical examination, routine blood investigations, and radiologic investigations. The recipient's vessels were evaluated with Doppler ultrasound (US) in all cases, and computed tomography (CT) angiography scan was performed in selected patients based on the clinical and/or Doppler US findings.

**Figure 1:**
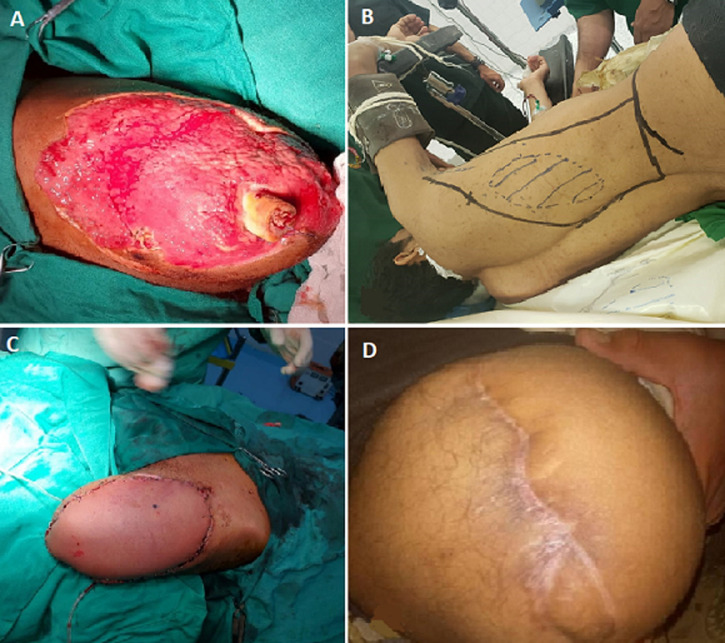
latissimus dorsi myocutaneous flap: A) the site of soft tissue defect with exposed bone; B) the latissimus dorsi myocutaneous flap preparation; C) the wound after flap coverage; D) show the results of flap coverage after ten months

**Figure 2:**
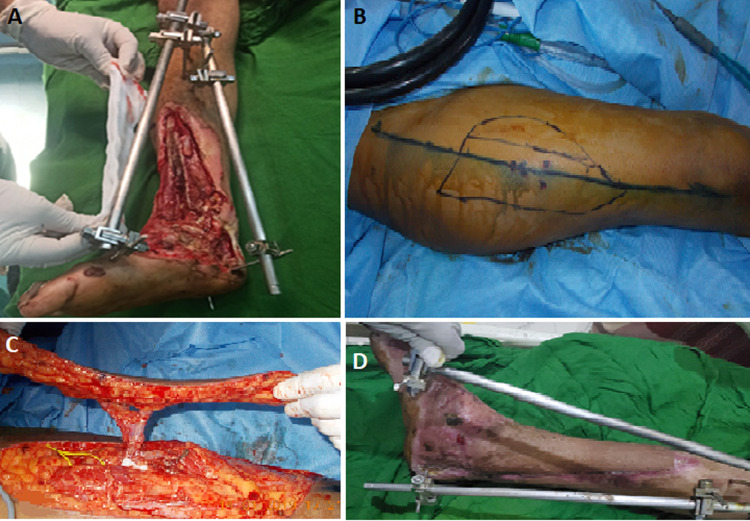
anterolateral thigh free flap: A) the site of soft tissue defect with exposed bone and joint; B) the anterolateral thigh flap design and preparation; C) the flap after dissection; D) the results of flap coverage after four weeks

**Figure 3:**
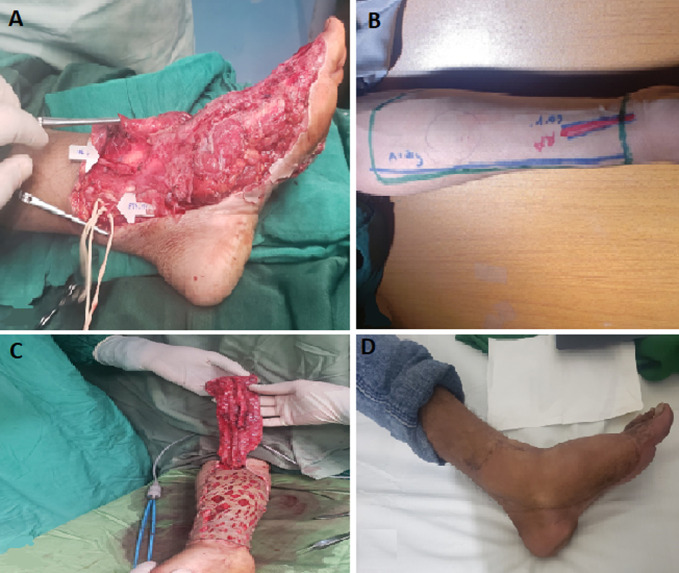
radial forearm free flap: A) the site of soft tissue defect with exposed bone; B) the radial forearm free flap design and preparation; C) the flap after dissection; D) the results of flap coverage after three months

**Figure 4:**
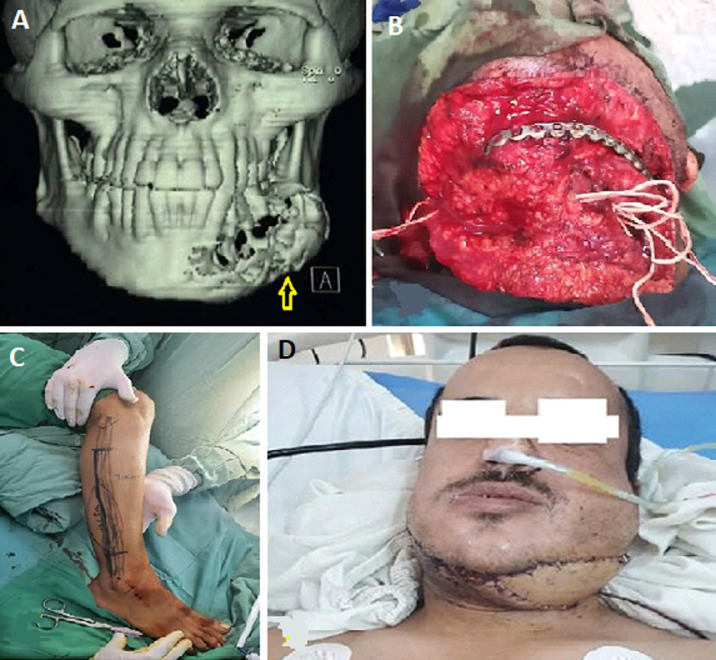
osteocutaneous fibula free flap: A) 3D computed tomography scan showing bony and soft tissue defects in the lower jaw (arrow); B) the intraoperative defect in the lower jaw; C) the fibula-free osteocutaneous flap design and preparation; D) the results of flap coverage after three weeks

**Operative techniques:** different surgical options, including free flap, were discussed with patients and their families, and written consent was obtained. The flaps were selected based on the defect characteristics and the surgeon's experience. All flaps were harvested and repaired under loupe magnification between 3.5 - 4.0x or operative microscope (Leica M530 OHX with glow technology ULT530, Leica Microsystems). Freestyle flaps are harvested in a random order after the perforator was visually identified by Doppler signals in a specific region [10,11]. Five types of flaps were used in our patients: latissimus dorsi free flap (Figure 1), anterolateral thigh flap (Figure 2), radial forearms free flap (Figure 3), fibulae osteo-cutaneous free flap (Figure 4); and gracilis flap.

Surgery consisted of debridement of damaged tissue or total resection of tumoral, exposure of recipient's vessels, the harvest of the selected flap, microsurgical anastomoses, and flap insert. Microvascular anastomoses were performed using an end-to-end or end-to-side technique with a single stitch nylon 9/0 suture, depending on the number of apparent vessels, vessel quality, proximity to the defect, and pedicle length. Papaverine was used locally to avoid pedicular spasms, and drains were routinely applied [12].

**Postoperative care:** the postoperative care includes anticoagulant therapy with heparin for the fourth postoperative day, antibiotic treatment, ambulation of patients depending on the graft site, surgeon preference, and dressing change. Flap monitoring included clinical observation (capillary refill, congestion, color), surface temperature readings, Doppler US evaluation, and pinprick testing. All skin sutures were removed two weeks after the operation. The functional rehabilitation program was recommended for all patients in our rehabilitation units.

**Study outcome:** the flap survival or failure was determined as the primary outcome of this study, whilst operative time and postoperative complications, including wound infection, hematoma, and partial flap necrosis, were considered the secondary outcomes [12]. Flap loss that necessities re-operation during hospitalization was defined as a partial flap failure. In comparison, total flap failure was defined as flap loss that necessitated additional coverage procedures or resulted in amputation [12]. Flap reconstruction was grouped into immediate (within 48 hours) or early (within 72 hours to 14 days) of trauma [2].

**Statistical analysis:** the mean ± SD, median described the quantitative variables, and for qualitative variables, frequency (percent) was used. Statistical analysis was performed using SPSS version 18 (SPSS Inc., Chicago, Illinois, USA).

**Ethical approval:** the study received ethics approval from 21 September University, Sana´a, Yemen, and the Medical Research Ethics Committee. All eligible participants were informed about the aims of the study; consent was signed before participation.

## Results

The baseline characteristics of the participants were mentioned in Table 1. The main age was 34.76 ± 16.88 years (range 10-67 years), with 24 (80%) men and 6 (20%) women. The most common indication (36.6%) was to replace extensive tissue loss caused by road traffic accidents, followed by post-tumor resection (30.0%). Most defects were in the lower extremities (50.0%), particularly the legs (23.3%). Ten cases were acutely managed (reconstruction was carried out in the same sitting of tumor resection or first wound debridement and management), and 20 patients had time intervals between injury time and microsurgical procedures (24 hours up to 4 weeks). In addition, 25 (83.3%) patients were managed with microsurgical free flaps as the primary option, and 5 (16.7%) patients shifted to microsurgery after the failure of the pedicle flap. The mean of defects required to be reconstructed was 84.9 ± 44.70 cm^2^(range 36 -220 cm^2^). The fibulae osteo-cutaneous free flap (30.0%), radial forearms free flap (23.3%), and anterolateral thigh flap (23.3%) were used most commonly. The main operative time was 7.09 ± 1.66 hours (range 4-11 hours).

**Table 1:** baseline characteristics of patients

Variables	N (%)
Age (years)	34.76 ± 16.88 (range 10-67)
**Gender**	
Male	24(80%)
Female	6(20%)
**Causes**	
Tumor	9(30.0%)
Weapon injury	7(23.3%)
Road traffic accidents	11(36.6%)
Work-related injuries	2(6.6%)
Electrical burn	1(3.3%)
**Site of defect**	
Head and neck	8(26.6%)
**Lower extremity**	
Thigh	1(3.3%)
Leg	7(23.3%)
Foot	4(13.3%)
Both leg and foot	3(10%)
**Upper extremity**	
Upper arm	3(10.0%)
Forearm	2(6.6%)
Hand	1(3.3%)
Trunk	1(3.3%)
Size of defect	84.9 ± 44.70 cm^2^ (range 36 -220)
**Indication of flap**	
Primary option	25(83.3%)
After pedicle flap failure	5(16.7%)
**Time of reconstruction**	
Immediate (0-48 h)	10(33.3%)
Early (72 h to 14 days)	20(66.7%)
**Flap use**	
Radial forearms free flaps	7(23.3%)
Latissimus dorsi free flaps	6(20.0%)
Anterolateral thigh flaps	7(23.3%)
Fibulae osteo-cutaneous free flaps	9(30.0%)
Gracilis flap	1(3.3%)
Operative time (hours)	7.09 ± 1.66 hours (range 4-11)
Mean follow-up time (months)	15.21 ± 4.64 (range 9-25)

The overall flap success rate was 83.3%. The total complication rate was 23.3%. Postoperative infection and partial flap necrosis occurred in 3 (10.0%) and 2 (6.6%), respectively. A total flap loss occurred in 5 (16.7%) patients and occurred in free osteocutaneous fibula flap in 3 cases (3 (10.0%) due to venous thrombosis and 2 (6.6%) due to arterial occlusion) (Table 2).

**Table 2:** postoperative complications and outcome of patients

Variables	N (%)
Total complication	7(23.3)
Partial necrosis	2(6.6%)
Wound dehiscence	2(6.6%)
Infection	3(10.0%)
**Outcome**	
Success	25(83.3%)
Failure	5(16.7%)
**Causes of flap failure**	
Venous thrombosis	3(10.0%)
Arterial occlusion	2(6.6%)

## Discussion

Reconstructive microsurgical techniques with a free flap for restoring severe soft tissue defects has shown to be an effective method for organ preservation and optimal functional result [13]. Our study indicates that flap use was associated with a high survival rate and acceptable complication rates; these findings go in line with other international outcome reports such as Barrette *et al*. and Alam *et al*. [14,15].

The use of free flap transfer was first described in 1973 when Daniel and Taylor used microscopic anastomosis to transfer a free groin flap. Similarly, a musculocutaneous free gracilis flap was used by Harii *et al*. in 1976. Subsequently, the use of free flaps has risen in clinical settings. Furthermore, the advancement of microsurgical techniques has led to widespread use with better outcomes [16,17]. Microvascular-free flap reconstruction of complex wounds is the highest part of the reconstruction surgery hierarchy and may be the last option for organ salvage. The outcomes can be optimized with a thorough preoperative assessment, adequate management of the wounds, and comorbid medical conditions. Such measures may enhance graft success and minimize the likelihood of reintervention, flap complications, or failure [3,8]. Moreover, satisfactory surgical approaches and equipment contribute to an increased flap survival rate. Fortunately, free flap transfer has a survival rate greater than 95% throughout many microsurgical institutions [18].

In our study, road traffic accidents (RTA) were the leading cause of soft tissue defects (36.6%), followed by defects due to tumor resection (30%), weapon injury (23.3%), work-related injuries (6.6%), and electrical burn injury (3.3%). Similarly, RTA was the most frequent trauma-related type of injury in the study by Alam *et al*. occurring in (50%) of patients, followed by blast injuries in 33% of cases, gunshot wounds in 10%, and crush injury in 10% of cases [15]. A recent study in Italy showed that tumor-associated defects were the most common cause (65.8%) preceding trauma (13.5%) [19]. The heterogeneity of the mechanism of the defect in various studies is attributed to the different geographical and demographic nature of the populations. Half of the reconstructed defects in our study population involved the lower extremities and were located in the legs in (23.3%) of cases. Our results were similar to previous reports [8,20]. Compared to other sites, defects on lower extremities are often more significant in size and lack local tissue, making reconstruction difficult and increased postoperative complications [21].

Minimizing flap morbidity is one of the reconstructive flap transfer cornerstones; therefore, ensuring an adequate vascular supply is sufficient. Given the complexity and inter-individual variability of perforator flaps, preoperative vascular mapping is becoming a necessity before any intervention. Furthermore, this practice was endorsed and recommended by several authors [4]. Observed promising results with the use of Doppler US in evaluating the vascular anatomy of free flaps, which overperformed CT scans [22]. In our study, we performed color Doppler US in all patients and CT angiography in selected cases preoperatively based on the clinical or US evaluation. The most suitable perforator flaps were identified and located depending on Doppler US results. Thereafter, the perforators' path in the corresponding area is determined based on the surgeon's assessment; such measures are aimed to reduce muscle and deep fascia incisions during the procedure and lower the complication rate [22].

In our study, only 10 (33.3%) flaps were done immediately within the first 48 hours post-trauma and early reconstruction with free flaps was performed in most cases (66.7%). It has been reported that a suitable result can be attained if primary wound coverage is provided concurrently with the use of free vascularized tissue transfer [15]. A recent meta-analysis that included 44,031 flaps reported a far better outcome with early flap transfer and close monitoring [23]. The optimal time for reconstructions is attributed to several factors such as soft tissue defect status (presence of edema, infection, or exposed neurovascular bundle). Reconstruction during the subacute stages was associated with a lower infection rate and other flap-related adverse events, allowing frequent debridement. The presence of fresh granulation tissue may indicate the optimal time for reconstruction, typically 2 to 3 days after the insult [12,24].

In our study, the defect size was 84.9 ± 44.70 cm^2^ (range 36-220) and was comparable to other reports [4]. Data regarding the effect of size defects on the final outcome are controversial. A recent systematic review found that the defect size did not affect the success rate or complication rate of flap reconstruction [21]. On the contrary, Zhang *et al*. reported that a flap size greater than 50 cm^2^ was associated with a higher postoperative complication rate [25]. Considerable perforator flap complications may be related to inadequate vascular supply; such an issue is problematic to many surgeons. Maintaining the donor site's aesthetic and normal function is also a struggle when a vast perforator flap is required. A large pedicled perforator flap donor site may be aesthetically acceptable in older patients with sagging skin; however, it may be unsightly in younger patients [12,25]. When the flap size is greater than 50 cm^2^, it is recommended that surgeons consider different treatment modalities, their experience, and the holistic assessment of the patient's condition.

In our study, the fibulae osteo-cutaneous free flaps were the most utilized flap (30%), followed by radial forearms free flaps (23.3%) and anterolateral thigh flap (23.3%). In the Bajec study, the latissimus dorsi musculocutaneous flap was used most frequently (52.4%), followed by the latissimus dorsi muscle flap (27.7%) in patients [20]. The eventual soft tissue coverage is attributed to several factors such as the defects' size and location, the injury's complexity, the surrounding tissue's condition, the exposure of vital structures, the patient's health status, microsurgical experience, and surgeon preference [26]. Thus, meticulously considering flap selection based on patient characteristics and presentation.

The mean operative time in our study was 7 hours, comparable to the previous report [13]. It was reported that prolonged operative time (> 10 hours) is linked with higher surgical adverse events [27,28]. In this study, the overall success rate of our intervention was 83.3%, and the failure rate was 16.7%. Wettstein *et al*. reported a 96% and 20% success and re-exploration rate in 197 patients with free flap reconstructions of the lower extremity, respectively [12]. Moreover, Melissinos *et al*. reported an almost similar success rate of 96.4%, with a slightly lower incidence of re-exploration of 14.7% [8]. The reported flap failure rate in Bajec *et al*. study was 11.88% [20]. Our failure rate was relatively higher than in previous studies. Several factors may have contributed to the failure rate found in our trials, including smaller sample size, surgeon expertise with this technique, variation in the type of flap utilized, mechanism of injury, and insufficient postoperative care, all of which might impair flap survival. More research is required to identify the causes of a greater prevalence of complications and reexploration.

In our study, the total rate of complications was 23.3%, and infection was present in 10%, which was successfully treated with a proper antibiotic, followed by partial necrosis in 6.6% and wound dehiscence in 6.6%, which was successfully treated with conservative treatments. Our result was in line with Kang *et al*. study [29]. In Qian *et al*. study, the complication rate was 13.5%, and partial flap necrosis was the most frequent postoperative complication, accompanied by temporary venous congestion, wound dehiscence, whole flap necrosis, hematoma formation, and arterial insufficiency [21].

In our study, the flap loss occurred in 16.7% and mainly emerged in free osteocutaneous fibula flap in 3 cases. The fibula flap has been used extensively for oromandibular reconstruction. Venous thrombosis, vessel kinking, and spasms have been reported as common causes of total free-flap failure in the early phase following microvascular anastomosis [30]. In our study, most osteocutaneous fibula flaps were utilized for post-cancer resection, and prior chemotherapy, radiotherapy, and nutritional status may contribute to more failure [31]. Another possibility for total flap loss is trauma during the fibula graft preparation, segmentation, and shaping [32].

In our study, the leading cause of flap failure was venous thrombosis and arterial occlusion. Similar reasons were reported by Bajec *et al*. [20]. Dependence on the limb, tight circumferential dressings, blood-caked dressings causing compression, and tracheostomy tapes around the neck are all factors that contribute to venous congestion in the flap [30].

The current study had several limitations. The retrospective nature and the small number of participants limited us from making a robust statistical analysis. Factors such as physical activity, economic status, skeletonization of perforators, pedicle rotation, comorbidity conditions (diabetic mellites, obesity, hypertension), and fracture healing may influence success rate and complication rate are not gathered. The duration of postoperative follow-up was short. We could not estimate the long-term effect of surgery. A prospective study with a large number and extended postoperative follow-up is recommended.

## Conclusion

Reconstruction of complex wounds with microsurgical free flaps is a reasonable option, even in a resource-limited setting. Despite many limitations, microsurgical free flaps in our setup were successful in most cases. In our study, the most common indication for replacing extensive tissue loss was caused by road traffic accidents. The most commonly used flaps are the fibulae osteo-cutaneous free flap, followed by radial forearms free flap and anterolateral thigh flap. Postoperative infection and partial flap necrosis were the most occurred complications.

### What is known about this topic

- *While reconstruction of complex wounds with severe tissue defects has been a significant problem in plastic surgery, free flap microsurgical procedures could solve many of these problems;*
- *Reconstruction of complex wounds with microsurgical free flaps is a viable option, even in a resource-limited setting*.

### What this study adds

- *This study provides information on microsurgery experiences in repairing complex wounds using different free flaps in Yemen;*
- *Despite many limitations, microsurgical free flaps were effective in treating individuals operated on in our setup*.
